# Hepatitis C virus (HCV) incidence among men who have sex with men (MSM) living with HIV: results from the French Hospital Database on HIV (ANRS CO4-FHDH) cohort study, 2014 to 2017

**DOI:** 10.2807/1560-7917.ES.2021.26.38.2001321

**Published:** 2021-09-23

**Authors:** Mathieu Castry, Anthony Cousien, Jonathan Bellet, Karen Champenois, Gilles Pialoux, Yazdan Yazdanpanah, Dominique Costagliola, Sophie Grabar, Sylvie Deuffic-Burban, on behalf of the French Hospital Database on HIV (ANRS CO4-FHDH Cohort)

**Affiliations:** 1 Université de Paris, INSERM, IAME, Paris, France; 2 Sorbonne Université, INSERM, Institut Pierre Louis d’Épidémiologie et de Santé Publique (IPLESP), Paris, France; 3 Sorbonne Université, Department of Infectious Diseases, APHP, Hôpital Tenon, Paris, France; 4 Service de maladies Infectieuses et tropicales, Hôpital Bichat Claude Bernard, Paris, France; 5 Assistance Publique-Hôpitaux de Paris (AP-HP), Département de Santé Publique, Hôpital Saint-Antoine, Paris, France; 6 The members of the ANRS CO4-FHDH cohort are acknowledged at the end of the article

**Keywords:** HCV incidence rate, HIV, MSM, DAA, ANRS CO4-FHDH, cohort study

## Abstract

**Background:**

Despite the availability of highly effective direct-acting antivirals (DAAs) and the expected treatment as prevention (TasP) effect, transmission of hepatitis C virus (HCV) persists in men who have sex with men (MSM) who engage in high-risk sexual behaviours.

**Aim:**

We aimed to estimate the incidence of primary HCV infection among MSM living with HIV in France when DAA was readily available.

**Methods:**

We used data from a large French hospital cohort of persons living with HIV (ANRS CO4-FHDH) prospectively collected between 2014 and 2017. HCV incidence rates were calculated using person-time methods for HCV-negative MSM at inclusion who had serological follow-up from 1 January 2014 to 31 December 2017. Sensitivity analyses were performed by varying the main assumptions to assess their impact on the results.

**Results:**

Of 14,273 MSM living with HIV who were initially HCV-seronegative, 330 acquired HCV during follow-up over 45,866 person-years (py), resulting in an overall estimated incidence rate of 0.72/100 py (95% CI: 0.65–0.80). HCV incidence significantly decreased from 0.98/100 py (95% CI: 0.81–1.19) in 2014 to 0.45/100 py (95% CI: 0.35–0.59) in 2017 (54% decrease; 95% CI: 36–67). This trend was confirmed by most of the sensitivity analyses.

**Conclusion:**

The primary incidence of HCV was halved for MSM living with HIV between 2014 and 2017. This decrease may be related to unrestricted DAA availability in France for individuals living with HIV. Further interventions, including risk reduction, are needed to reach HCV micro-elimination in MSM living with HIV.

## Introduction

Since 2000, hepatitis C virus (HCV) infection has emerged as an epidemic among men who have sex with men (MSM) living with HIV, especially in high-income countries [1-3]. Using data from the international CASCADE collaboration, van Santen et al. found that HCV incidence among MSM living with HIV significantly increased between 1990 and 2014, although there were some geographical variations within Europe [4]. Transmission of HCV in this population has been associated with high-risk practices such as unprotected traumatic sex (including fisting, which may cause blood contact), group sex, and drug-use behaviours [5-8]. Moreover, as acute HCV infections were mainly found among MSM living with HIV, HIV infection might facilitate sexual transmission of HCV [9]. As persons living with HIV (PLHIV) experience accelerated liver disease progression [10], early HCV treatment has been recommended [11,12].

The advent of highly effective, direct-acting antiviral (DAA) therapy for HCV, which is well tolerated, has a shorter treatment duration, and a sustained viral response (SVR) that exceeds 90% in both HCV monoinfected and HIV/HCV coinfected populations [13,14] raises the possibility of HCV elimination. In this context, the World Health Organization (WHO) has issued HCV elimination targets that include a 90% reduction in HCV incidence by 2030 (relative to 2015) [15], a target that France has set for 2025 [16]. In France, from 2014 to mid-2016, the use of second-generation DAAs, which are particularly effective and well tolerated, was restricted to HCV-infected patients with severe fibrosis except in patients with comorbidities (including HIV coinfection) regardless of fibrosis stage. A study published in 2019 reported that DAA scale-up increased sooner among PLHIV, especially between 2014 and 2015 (89% increase in the number of PLHIV using DAA therapy) [17].

In this favourable context, it is interesting to assess the trend of HCV incidence to determine the impact of DAAs on the epidemic and to estimate the potential for improvement. In view of this and to better inform public health strategies, this study estimates the incidence of primary HCV infection among MSM living with HIV enrolled in the French Hospital Database on HIV (ANRS CO4-FHDH) between 2014 and 2017 (i.e., the DAA era).

## Methods

### Study population

We used data from the ANRS CO4-FHDH – a nationwide open cohort created in 1989 – to follow PLHIV receiving care in 175 public hospitals located throughout France. The ANRS CO4-FHDH cohort is representative of the PLHIV population receiving medical care in France [18]. Individuals were included in the ANRS CO4-FHDH cohort if they had a documented HIV-1 or HIV-2 infection and were followed-up in a centre participating in the FHDH [18]. Standardised variables are collected at each outpatient visit or hospital admission when a new clinical manifestation is diagnosed, a new treatment is prescribed, or a change in biological markers is noted at least once over 6 months.

In the present study, incident HCV infection was assessed among men who met the following criteria: (i) recorded as having acquired HIV through sex between men (regardless of injecting drug use); (ii) at least one clinic visit between 2014 and 2017; (iii) with a baseline negative anti-HCV test in the 2 years preceding the date of inclusion in the analysis; and (iv) at least one subsequent HCV test between 2014 and 2017. Individuals were considered lost to follow-up (LTFU) if they had no clinic visit for 18 months [19].

### HCV primary incidence calculation

Follow-up time was calculated from 1 January 2014, cohort enrolment, or last clinic visit preceding the first negative anti-HCV test (for individuals with unknown HCV status at inclusion). Follow-up duration was calculated until death, LTFU, 31 December 2017, or the date of HCV infection (see details below), whichever occurred first.

Incident HCV infection was based on any positive HCV test (RNA and/or antibodies) during follow-up. Only the first observed HCV infection was included in the analysis. In France, current guidelines for MSM living with HIV recommend HCV screening at least once every 6 months [20]. Based on this information and assuming this testing pattern did not change during the study period, the date of HCV infection among MSM who acquired HCV during follow-up was estimated as the midpoint between the last HCV negative test and first HCV positive test, when the test interval was 6 months or less. If the test interval was more than 6 months, we assumed that the date of infection was the date of first positive HCV test minus 3 months (i.e., half the recommended testing interval). HCV negative status was assigned to individuals who had at least one HCV negative test and who never tested HCV positive throughout follow-up, which ended on 31 December 2017. This strategy assumed that negative HCV tests were not systematically reported due to the nature of the data (i.e., data were recorded in a hospital database). Therefore, we assumed that individuals were still at risk as long as they were regularly followed in the ANRS CO4-FHDH cohort. For patients LTFU, follow-up stopped 6 months after last clinic visit before LTFU.

The incidence rate was calculated as the number of incident infections divided by the number of person-years (py) at risk to acquire HCV. We estimated overall and yearly HCV primary infection incidence rates between 2014 and 2017 and calculated 95% confidence intervals (95% CI) using exact Poisson confidence limits. We used Poisson regression to test for the trend in HCV incidence over calendar-time. Additional analyses were performed to estimate the incidence rates with the following characteristics: age at inclusion (< 30, 30–45, > 45 years) and region of residence (Paris area (Ile-de-France) or other).

### Sensitivity analyses

We performed six sensitivity analyses to assess the impact of our main assumptions on incidence rate estimations.

As the interval between the last HCV negative and first HCV positive test could have been long, the date of infection could not be determined with certainty. Therefore, we considered two situations in the main analysis: (i) test interval lower or (ii) higher than 6 months. To assess the impact of our hypothesis, we started by conducting the following two sensitivity analyses:

- sensitivity analysis 1: date of infection = the date of first HCV positive test regardless of the test interval; and
- sensitivity analysis 2: date of infection = midpoint between the last negative and first positive HCV test regardless of the test interval.

Next, we included individuals LTFU 6 months after their last clinic visit before LTFU in the main analysis and conducted the following two sensitivity analyses:

- sensitivity analysis 3: end of follow-up = 12 months after last clinic visit before LTFU; and
- sensitivity analysis 4: end of follow-up = date of last clinic visit before LTFU.

Finally, as the incidence rate is highly sensitive to the number of py at risk to acquire HCV, we assessed two ending dates for HCV negative individuals with continued follow-up in the ANRS CO4-FHDH cohort (cf. 31 December 2017 in the main analysis):

- sensitivity analysis 5: end of follow-up = date of last clinic visit; and
- sensitivity analysis 6: end of follow-up = date of last negative HCV test.

Supplementary Figures S1, S2 and S3 provide graphical illustrations of the assumptions used in the different analyses and their impact on follow-up time estimation. The analyses were performed using SAS version 9.4 software (SAS Institute, Cary, North Carolina, United States).

### Ethical statement

The ANRS CO4-FHDH cohort was approved by the French data protection authority (CNIL – Commission Nationale de l’Informatique et des Libertés) on 27 November 1991 (Journal Officiel, 17 January 1992). All volunteers gave written informed consent to participate.

## Results

**Figure 1:**
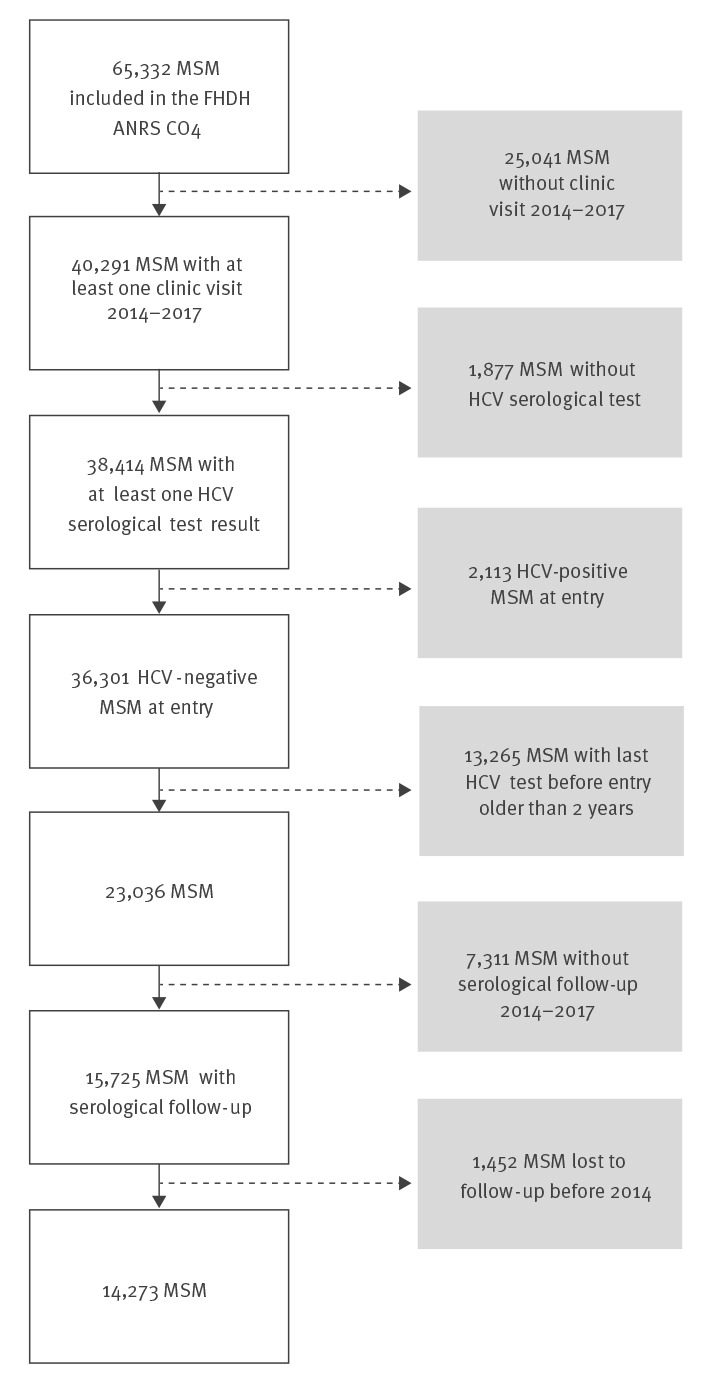
Flow diagram of the study population selection for the HCV incidence analysis, France, 2014–2017 (n = 14,273)

Since 1989, the ANRS CO4-FHDH cohort has included 65,332 MSM living with HIV. Of these, 40,291 had at least one clinic visit between 2014 and 2017 and 38,414 (95%) had at least one anti-HCV test result (Figure 1), of which 2,113 (5.5%) were HCV-infected at study entry. Of the remaining 36,301 study participants HCV-seronegative at inclusion, 13,265 were excluded because their HCV-seronegative test was considered out of date (> 2 years old). At least one serological result was available between 2014 and 2017 for 15,725 of the initially HCV-negative study participants. Finally, after excluding those LTFU before 2014, a total of 14,273 MSM living with HIV were included in the analysis. Characteristics of the participants are described in Table 1. Their median age was 44 years (Interquartile range (IQR): 35–52). Individuals received an HCV serology a median of 3 (IQR: 2–4) times between 2014 and 2017, with a median testing interval of 11.6 (IQR: 7.7–16.1) months.

ANRS CO4-FHDH: French hospital database on HIV; HCV: hepatitis C virus; MSM: men who have sex with men.

The grey boxes depict MSM whose data were excluded from the analysis.

**Table 1:** Main characteristics of the MSM living with HIV from the ANRS CO4-FHDH cohort included in the present analysis, 2014–2017 (n = 14,273)

Characteristics	Total (n = 14,273)
Age at inclusion in the analysis (years)	44 (IQR: 35–52)
Time between HIV diagnosis and inclusion in the analysis (years)	6.5 (IQR: 1.4–16.8)
*HIV transmission group*	
MSM	14,198 (99.5%)
MSM + PWID	75 (0.5%)
*HIV serology*	
HIV-1	14,251 (99.8%)
HIV-2	8 (0.1%)
HIV-1 and HIV-2	14 (0.1%)
Antiretroviral treatment at inclusion in the analysis^a^	11,914 (83.5%)
Antiretroviral treatment at the end of follow-up^b^	14,117 (98.9%)
AIDS before inclusion in the analysis	2,464 (17.3%)
HIV viral load < 50 copies/ml at inclusion in the analysis^c^	9,569 (71.6%)
CD4+ cell count (cells/μl) at inclusion in the analysis^d^	609 (IQR: 441–798)

AIDS: acquired immunodeficiency syndrome; ANRS CO4-FHDH: French hospital database on HIV; IQR: interquartile range; MSM: men who have sex with men; PWID: people who inject drugs.

^a^ Treatment initiation occurring before the date of inclusion in the analysis.

^b^ Treatment initiation occurring before the end of follow-up in the analysis.

^c^ Last viral load measure in the 12 months before the date of inclusion in the analysis. Data available for 13,361 patients (93.6%).

^d^ Last Lymphocyte T CD4 measure in the 12 months before the date of inclusion in the analysis. Data available for 13,501 patients (94.6%).

Data are presented as count (percentage) or median (interquartile range).

### Hepatitis C virus incidence

**Figure 2:**
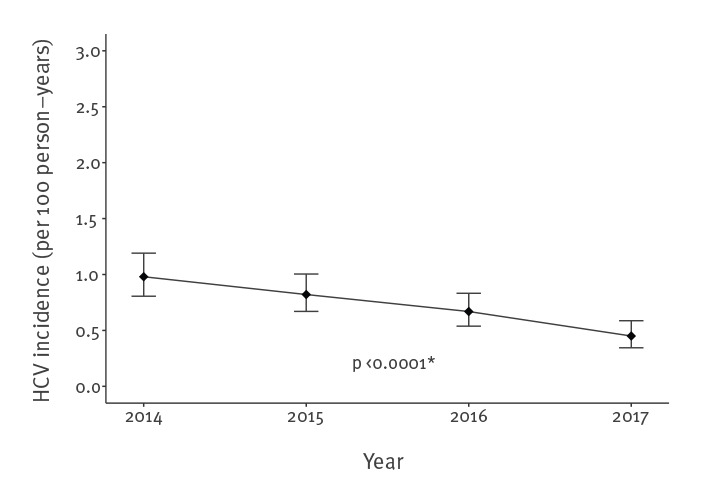
Hepatitis C virus incidence among MSM living with HIV from the ANRS CO4-FHDH cohort. France, 2014–2017 (n = 14,273)

These MSM living with HIV accounted for a total of 45,866 py of follow-up with a median follow-up time of 4 years (IQR: 2–4). With 330 new incident HCV infections, the estimate of the overall incidence rate was 0.72/100 py (95% CI: 0.65–0.80). The absolute number of new infections continuously declined over time: 101 in 2014, 94 in 2015, 81 in 2016, and 54 in 2017. Annual incidence trends are presented in the first part of Table 2 and in Figure 2. Incidence rates of first HCV infections significantly decreased (Poisson regression, p trend < 0.0001), from 0.98/100 py (95% CI: 0.81–1.19) in 2014 to 0.45/100 py (95% CI: 0.35–0.59) in 2017, a 54% decrease (95% CI: 36–67).

**Table 2:** Main analysis and sensitivity analyses of hepatitis C virus incidence among MSM living with HIV from the ANRS CO4-FHDH cohort, by year and overall, France, 2014–2017 (n = 14,273)

Year	Person-years	Number of infections	Incidence/100 py (95% CI)	Trend (p value)
**Main analysis**				
2014	10,308	101	0.98 (0.81–1.19)	p < 0.0001
2015	11,455	94	0.82 (0.67–1.00)	
2016	12,100	81	0.67 (0.54–0.83)	
2017	12,003	54	0.45 (0.35–0.59)	
2014–2017	45,866	330	0.72 (0.65–0.80)	
**Sensitivity analysis 1: date of infection = date of first positive HCV test^a^**				
2014	10,330	104	1.01 (0.83–1.22)	p < 0.0001
2015	11,474	97	0.85 (0.69–1.03)	
2016	12,118	86	0.71 (0.57–0.88)	
2017	12,015	67	0.56 (0.44–0.71)	
2014–2017	45,938	354	0.77 (0.69–0.86)	
**Sensitivity analysis 2: date of infection = midpoint between the last negative and first positive HCV test^a^**				
2014	10,273	114	1.11 (0.92–1.33)	p < 0.0001
2015	11,414	86	0.75 (0.61–0.93)	
2016	12,076	65	0.54 (0.42–0.69)	
2017	11,994	37	0.31 (0.22–0.43)	
2014–2017	45,757	302	0.66 (0.59–0.74)	
**Sensitivity analysis 3: end of follow-up for LTFU patients = 12 months after last clinic visit before LTFU^b^**				
2014	10,538	102	0.97 (0.80–1.18)	p < 0.0001
2015	11,657	95	0.81 (0.67–1.00)	
2016	12,376	81	0.65 (0.52–0.81)	
2017	12,094	54	0.45 (0.34–0.58)	
2014–2017	46,665	332	0.71 (0.64–0.79)	
**Sensitivity analysis 4: end of follow-up for LTFU patients = date of last clinic visit before LTFU^b^**				
2014	10,082	101	1.00 (0.82–1.22)	p < 0.0001
2015	11,215	94	0.84 (0.69–1.03)	
2016	11,862	81	0.68 (0.55–0.85)	
2017	12,003	54	0.45 (0.35–0.59)	
2014–2017	45,162	330	0.73 (0.66–0.81)	
**Sensitivity analysis 5: end of follow-up for HCV negative patients with continued follow-up in the cohort = date of last clinic visit^c^**				
2014	10,272	101	0.98 (0.81–1.19)	p = 0.0008
2015	11,322	94	0.83 (0.68–1.02)	
2016	11,803	81	0.69 (0.55–0.85)	
2017	9,041	54	0.60 (0.45–0.77)	
2014–2017	42,437	330	0.78 (0.70–0.86)	
**Sensitivity analysis 6: end of follow-up for HCV negative patients with continued follow-up in the cohort = date of last negative HCV test^c^**				
2014	9,849	101	1.03 (0.84–1.25)	p = 0.4579
2015	9,848	94	0.95 (0.78–1.17)	
2016	8,627	81	0.94 (0.76–1.17)	
2017	4,279	54	1.26 (0.97–1.65)	
2014–2017	32,602	330	1.01 (0.91–1.13)	

ANRS CO4-FHDH: French hospital database on HIV; CI: confidence interval; HCV: hepatitis C virus; LTFU: Lost to follow-up; py: person years.

^a^ Date of infection (in the main analysis) = midpoint between the last negative and the first positive HCV test when the interval was ≤ 6 months, or the date of first positive HCV test minus 3 months when > 6 months.

^b^ End of follow-up for LTFU patients (in the main analysis) = 6 months after last clinic visit before LTFU.

^c^ End of follow-up for HCV negative patients with continued follow-up in the cohort (in the main analysis) = 31 December 2017.

ANRS CO4-FHDH: French hospital database on HIV; HCV: hepatitis C virus.

Whiskers represent 95% confidence intervals of the point estimate.

* p trend over 2014–2017 (Poisson regression).

**Figure 3:**
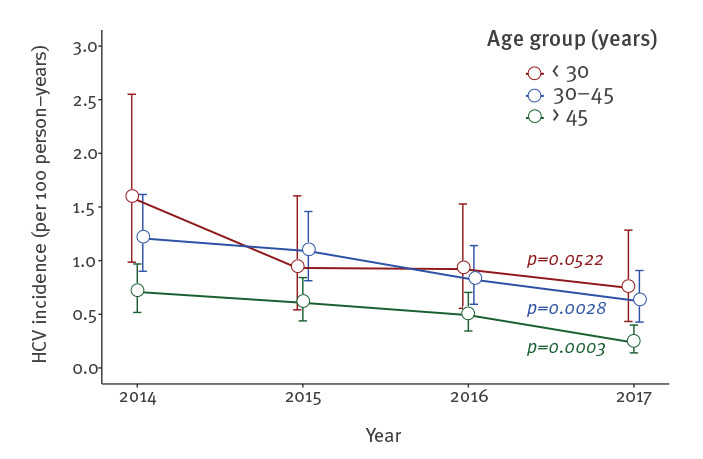
Hepatitis C virus incidence among MSM living with HIV from the ANRS CO4-FHDH cohort, by age group. France, 2014–2017 (n = 14,273)

**Figure 4:**
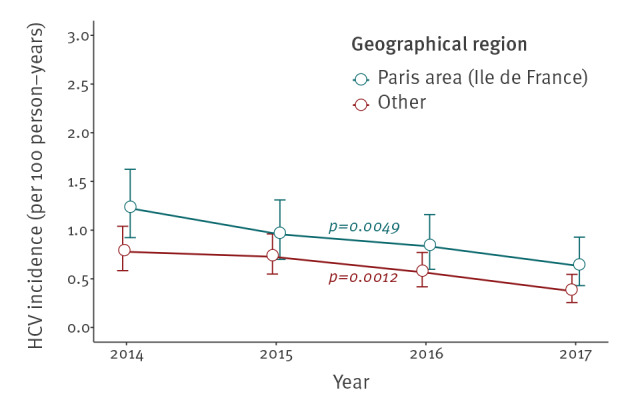
Hepatitis C virus incidence among MSM living with HIV from the ANRS CO4-FHDH cohort, by geographical region. France, 2014–2017 (n = 14,273)

Some differences were observed when stratifying by age or place of residence (Figures 3 and 4). Younger MSM were more likely to experience HCV infection, whether < 30 vs > 45 years (incidence rate ratio (IRR): 1.96; p value < 0.0001) or 30–45 vs > 45 years (IRR: 1.82; p value < 0.0001). HCV incidence decreased over time in all age groups, although this was not significant for those under 30 years of age at inclusion (p = 0.0522). Similarly, the HCV incidence rate was higher among those living in the Paris area compared with those living in other areas (IRR: 1.51; p value = 0.0003).

ANRS CO4-FHDH: French hospital database on HIV; HCV: hepatitis C virus; MSM: men who have sex with men.

Whiskers represent 95% confidence intervals of the point estimate.

p trend values over 2014–2017 were obtained with Poisson regression.

ANRS CO4-FHDH: French hospital database on HIV; HCV: hepatitis C virus; MSM: men who have sex with men.

Whiskers represent 95% confidence intervals of the point estimate.

p trend values 2014–2017 were obtained with Poisson regression.

### Sensitivity analyses

Results for the sensitivity analyses are presented in the second part of Table 2. Assuming the time of infection to be the date of first positive HCV test (sensitivity analysis 1), we observed similar results to those of the main analysis. However, there was one notable difference for the incidence rate in 2017: the main analysis revealed an incidence rate of 0.45/100 py, whereas sensitivity analysis 1 revealed an incidence rate of 0.56/100 py. In the sensitivity analysis using the midpoint between the last negative and first positive HCV test to estimate the date of infection (i.e., sensitivity analysis 2), we observed a lower overall incidence rate between 2014 and 2017 (0.66/100 py) compared with the main analysis incidence rate (0.72/100 py) and a more marked decline over time. Indeed, HCV incidence decreased from 1.11/100 py in 2014 to 0.31/100 py in 2017. Overall and annual estimates for HCV incidence changed slightly when assuming a different ending date for LTFU patients – i.e., 12 months after last clinic visit (sensitivity analysis 3) or at the date of last clinic visit before LTFU (sensitivity analysis 4). When follow-up was censored at the date of last clinic visit instead of 31 December 2017 for HCV negative individuals with continued follow-up in the cohort (sensitivity analysis 5), the overall incidence was slightly higher, mainly due to the incidence rate in 2017 (0.60/100 py), compared to the incidence rate in the main analysis (0.45/100 py). Finally, the greatest differences were found in the sensitivity analysis 6, which estimated end of follow-up for HCV negative individuals with continued follow-up as the date of last negative HCV test. Overall incidence rate was 1.01/100 py, which was much higher than for the main analysis. Annual incidence trend was stable (p trend = 0.46), decreasing slightly from 1.03/100 py in 2014 to 0.94/100 py in 2016 and then increasing to 1.26/100 py in 2017.

## Discussion

The present study estimates the incidence of HCV between 2014 and 2017 in a large cohort of MSM living with HIV in France. We observed a significant decrease in HCV primary incidence between 2014 and 2017, from 0.98 to 0.45/100 py. The most pronounced decrease in HCV incidence was observed over the last year (33% reduction between 2016 and 2017).

Our results coincide with scale-up of DAAs observed in France since 2014 among PLHIV [17]. A decrease in HCV incidence has been reported as a possible consequence of HCV treatment scaling-up in other settings. For example, in the Netherlands, DAAs became widely available in 2015, and two prospective studies among MSM living with HIV in 2014 and 2016 reported a 51% decrease in HCV incidence between 2014 and 2016 [21]. Interestingly, a study published in 2020 analysing phylogenetic HCV strains among HIV-infected MSM diagnosed with HCV subtype 4d during the 2003–2007 HCV outbreak highlighted the close links between the French and the Dutch MSM communities [22]. Additionally, a decreasing trend was observed among MSM living with HIV in the United Kingdom between 2013 and 2018 [23]. Our study shows similar trends, although we must be cautious with such comparisons because of differences in populations, cohort design and methods. Furthermore, we cannot fully rule out that the observed decrease in HCV incidence is due to changes in risk behaviour. However, a study published in 2018 found that inconsistent condom use with casual partners increased between 2000 and 2017 among HIV-infected MSM enrolled in the French ANRS PRIMO cohort [24]. Similar trends were observed for syphilis cases among HIV-infected MSM in the Netherlands [21] and Switzerland [25]. Therefore, the decrease in HCV incidence is unlikely to be the result of reduced sexual risk behaviours, although more evidence is needed, particularly on risk behaviours directly associated with HCV transmission in this population. Although treatment scale-up would produce further reductions in HCV incidence, reaching the WHO HCV elimination target would also require a commitment to risk reduction [26].

Pradat et al. [27] found an increase in HCV incidence between 2012 and 2016 among French MSM living with HIV using data from the Dat’AIDS cohort (a prospective cohort of PLHIV from a subset of 15 FHDH centres). We thus replicated our analyses after selecting patients followed in the Dat’AIDS centres (see Supplementary Table S1). Although the incidence appears more stable between 2015 and 2016, our overall conclusions remained unchanged, suggesting that the differences we obtained, compared with Pradat et al., are likely due to differences in methodological choices. Although Pradat et al. did not provide a detailed description of their analytical methodology, their analysis appears to be based on the assumptions that we tested in sensitivity analyses 1 (resulting in a higher number of new infections, especially in 2017) and sensitivity analyses 6 (resulting in a lower number of py, which decreased over time). In a meeting in December 2019, we informed Pradat et al. that our results differed from theirs and informed them of our intention to publish this article.

Several studies have observed high reinfection incidence rates among MSM living with HIV [21-24]. We did not estimate HCV reinfections as we lacked reliable data regarding HCV treatment, although this important issue needs to be addressed as HCV reinfections could sustain the epidemic. Indeed, one cannot exclude that some HCV treatments were initiated in hepatology units without being registered in our database. However, the global incidence is mainly driven by the primary incidence. Likewise, our analysis did not include HIV-negative MSM, including those receiving preexposure prophylaxis (PrEP) for whom high incidence of HCV has been reported in France [28,29]. For example, an acute HCV incidence rate of 1.40/100 py was found between 2012 and 2016 among at-risk HIV-negative MSM enrolled in the ANRS IPERGAY PrEP trial [30]. Given that both populations are closely related high-risk groups and share HCV transmission networks [31,32], this could impact the HCV epidemic among HIV-positive MSM and thereby halt the progress towards HCV elimination, a possibility that shows the need for strengthening secondary prevention interventions that sustain the decrease of HCV. However, estimates of HCV incidence trends among HIV-negative MSM would be needed.

Our study has a number of limitations. First, we used hospital-based data, which we know are imperfectly reported and not primarily collected for our purpose. Indeed, as with many recent cohorts, ANRS CO4-FHDH is a clinical cohort in which individuals are followed as they access hospital care. The timing and the type of data collected depends on patients’ care and not necessarily on the needs of research projects. That is, these cohort studies are unlike the classic interval cohort design studies where investigators predetermine the individuals to be followed at specified intervals regardless of their medical needs [33]. This difference introduces issues that need to be addressed in the analysis. Here, this was particularly the case for negative anti-HCV tests, which were likely under-reported given the clinical cohort design. To overcome this under-reporting, we had to make several assumptions, so we may have underestimated the incidence rate by assuming that individuals tested HCV-negative at least once during the study period and never tested HCV positive remained HCV negative as long as they had regular clinic visits. However, this assumption was based on existing guidelines regarding follow-up and HCV testing frequency among MSM living with HIV in France. Second, we cannot rule out a possible bias linked to heterogeneity in data reporting between centres. Third, our study has limited value for understanding disease aetiology as we lacked data on potential exposures, especially pertaining to risk behaviours related to sexual practices, use of drugs in a sexual context (e.g., chemsex and slamsex), and ongoing injecting drug use.

The main strength of our study is the large size of the study population and its representativeness. Moreover, we considered various sensitivity analyses to overcome the possible biases associated with the nature of the data to obtain valid and reliable estimates of HCV incidence. Our conclusions remained robust: five of six analyses showed a decline in HCV incidence over calendar periods (steeper in sensitivity analysis 2). Using the date of the last negative HCV test as the end of follow-up for HCV negative individuals (sensitivity analysis 6), which is closer to the method used by Pradat et al. [27], led to stable incidence estimates for 2014–2017 (p trend = 0.46). However, this assumption seems unlikely since negative tests may not be systematically reported in hospital-based data. This method leads to shorter follow-up time for MSM remaining HCV-negative throughout follow-up. Consequently, this approach is likely to overestimate HCV incidence and highlights the importance and the need to be thoughtful about the data reported as well as the data omitted.

## Conclusion

We observed a strong decrease in primary HCV infections among MSM living with HIV in France between 2014 and 2017. This decrease may be related to a concomitant and continuous scaling-up in DAA use, which was especially marked in HIV-HCV coinfected individuals. This decrease in HCV incidence could also be due to changes in risk reduction. Enhanced vigilance is required to monitor HCV incidence and to determine the prevention tools needed to reach micro-elimination of the HCV epidemic in this population.

## Supplementary Data

1001000 Supplementary Material 1
