# Supplementary material for: Hepatitis C virus (HCV) incidence among men who have sex with men (MSM) living with HIV: results from the French Hospital Database on HIV (ANRS CO4-FHDH) cohort study, 2014 to 2017
Source: Euro Surveill. 2021 Sep 23;26(38):2001321. doi: 10.2807/1560-7917.ES.2021.26.38.2001321 (PMC8462035; doi:10.2807/1560-7917.ES.2021.26.38.2001321)
Supplement: Supplementary Material 1 [file 20-01321_CASTRY_Supplementary_material.pdf]

Supplementary materials to the article:

Hepatitis C virus (HCV) incidence among men who have sex with men (MSM) living with HIV in France: results from the French Hospital Database on HIV (ANRS CO4-FHDH) cohort study, 2014 to 2017

This supplementary material is hosted by *Eurosurveillance* as supporting information alongside the article "*Hepatitis C virus (HCV) incidence among men who have sex with men (MSM) living with HIV in France: results from the French Hospital Database on HIV (ANRS CO4-FHDH) cohort study, 2014 to 2017*", on behalf of the authors, who remain responsible for the accuracy and appropriateness of the content. The same standards for ethics, copyright, attributions and permissions as for the article apply. Supplements are not edited by *Eurosurveillance* and the journal is not responsible for the maintenance of any links or email addresses provided therein.

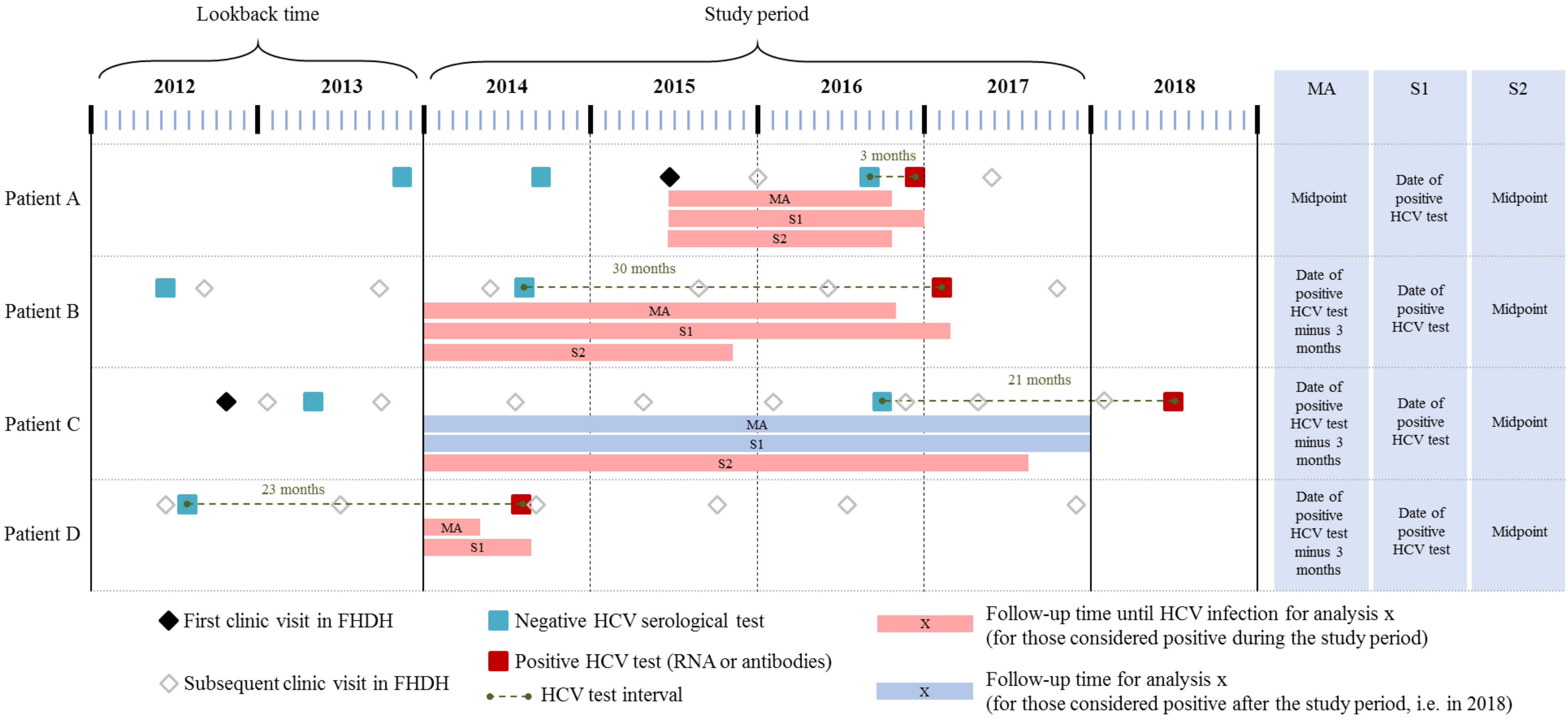

Supplementary Figure S1: **Illustration of different analyses to estimate the date of infection**.

Notes: Four hypothetical patients with a positive HCV test and a continued follow-up in the FHDH cohort (i.e if time interval between clinic visits was ≤ 18 months) are shown. The columns at right indicate the method used to estimate the date of infection according to the different analyses: main analysis (date of infection = midpoint between the last negative and the first positive HCV test when the interval was ≤ 6 months, or the date of first positive HCV test minus 3 months when > 6 months), sensitivity analysis 1 (date of infection = date of first positive HCV test) or sensitivity analysis 2 (date of infection = midpoint between the last negative and first positive HCV test). **Abbreviations**: MA = main analysis; S1 = sensitivity analysis 1; S2 = sensitivity analysis 2.

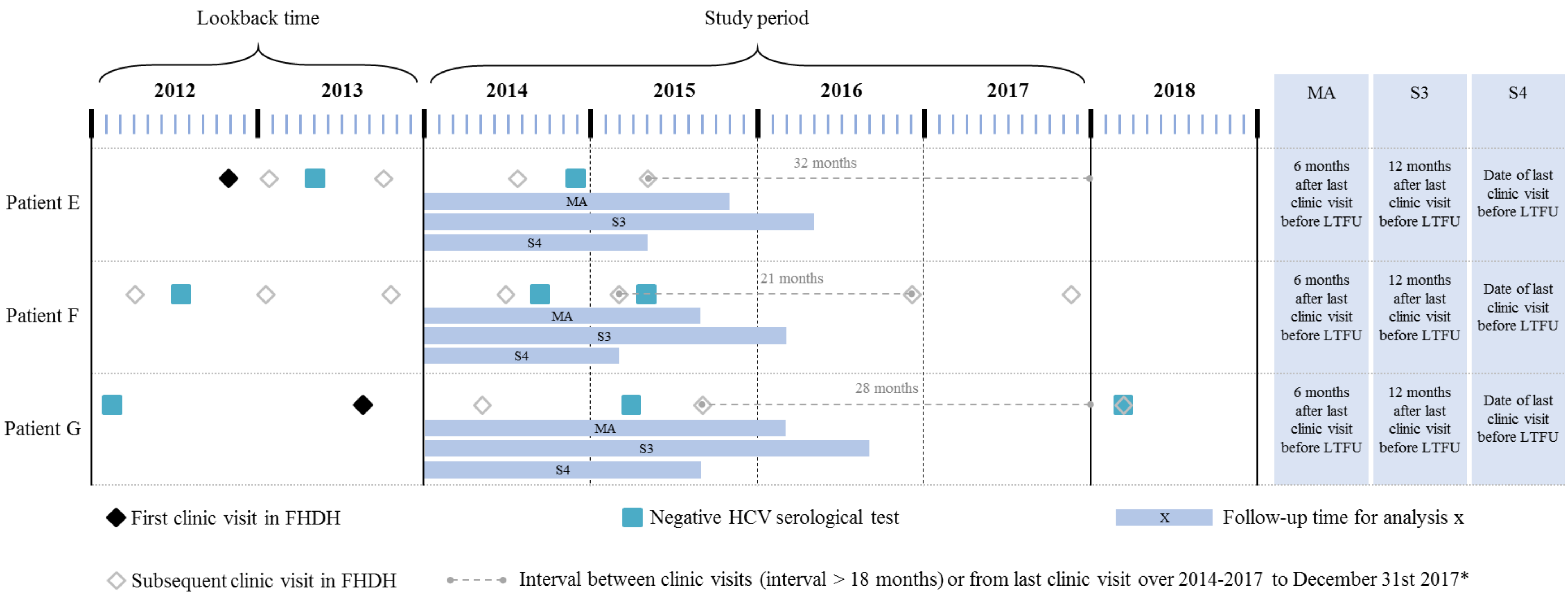

Supplementary Figure S2: Illustration of different analyses to estimate end of follow-up for lost to follow-up (LTFU) patients.

Notes: Three hypothetical patients with only negative HCV tests and LTFU (i.e if time interval between clinic visits or from last clinic visit to December 31st 2017 was > 18 months). The columns at right indicate the method used to estimate follow-up duration, according to the different analyses: main analysis (end of follow-up = 6 months after last clinic visit before LTFU), sensitivity analysis 3 (end of follow-up = 12 months after last clinic visit before LTFU) or sensitivity analysis 4 (end of follow-up = date of last clinic visit before LTFU). **Abbreviations**: MA = main analysis; S3 = sensitivity analysis 3; S4 = sensitivity analysis 4; LTFU=Lost to follow-up.

* If last database update for one center was before December 31st 2017, then censoring was made in regard to last update instead of December 31st 2017 (for a patient followed in that center).

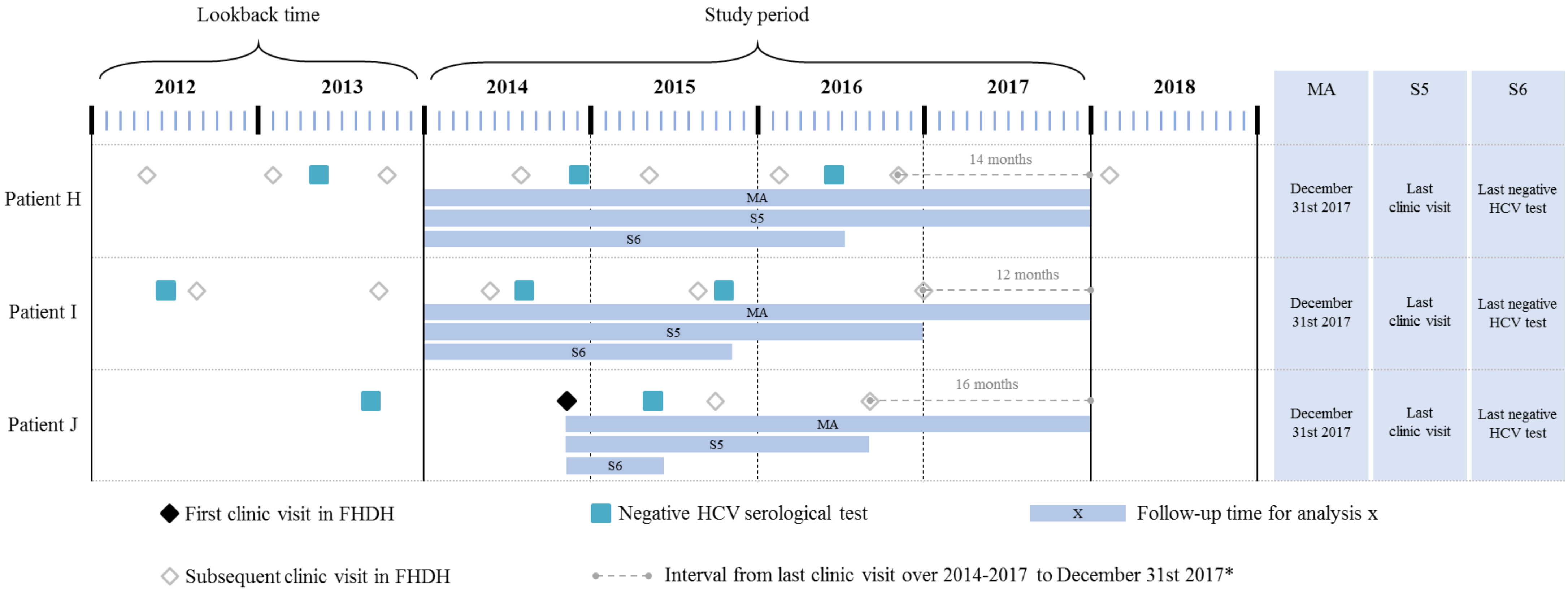

Supplementary Figure S3: Illustration of different analyses to estimate end of follow-up for HCV negative patients with continued follow-up in the cohort

Notes: Three hypothetical patients with only negative HCV tests and a continued follow-up in the cohort (i.e if time interval between clinic visits and from last clinic visit to December 31st 2017 was ≤ 18 months). The columns at right indicate the method used to estimate follow-up duration according to the different analyses: main analysis (end of follow-up = December 31st 2017), sensitivity analysis 5 (end of follow-up = date of last clinic visit) or sensitivity analysis 6 (end of follow-up = last negative HCV test). **Abbreviations**: MA = main analysis; S5 = sensitivity analysis 5; S6 = sensitivity analysis 6.

* If last database update for one center was before December 31st 2017, then censoring was made in regard to last update instead of December 31st 2017 (for a patient followed in that center).

Table S1. Hepatitis C virus incidence among MSM living with HIV from the ANRS CO4-FHDH cohort (selection on patients followed in the 15 Dat'AIDS centers), yearly and overall; main and sensitivity analyses. France, 2014-2017 (n=6,007 patients selected).

Year	Person-years	Number of infections	Incidence/100 py [95% CI]	Trend (p-value)
Main analysis				
2014	4,043	41	1.01 [0.75-1.38]	p=0.0037
2015	4,383	31	0.71 [0.50-1.01]	
2016	4,586	32	0.70 [0.49-0.99]	
2017	4,607	21	0.46 [0.30-0.70]	
2014-2017	17,619	125	0.71 [0.60-0.85]	
Sensitivity analysis 1: date of infection = date of first positive HCV test[a]				
2014	4,052	45	1.11 [0.83-1.49]	p=0.0108
2015	4,390	33	0.75 [0.53-1.06]	
2016	4,593	32	0.70 [0.49-0.98]	
2017	4,612	28	0.61 [0.42-0.88]	
2014-2017	17,647	138	0.78 [0.66-0.92]	
Sensitivity analysis 2: date of infection = midpoint between the last negative and first positive HCV test[a]				
2014	4,034	44	1.09 [0.81-1.47]	p=0.0001
2015	4,374	30	0.69 [0.48-0.98]	
2016	4,579	30	0.66 [0.46-0.94]	
2017	4,604	17	0.37 [0.23-0.59]	
2014-2017	17,591	121	0.69 [0.58-0.82]	
Sensitivity analysis 3: end of follow-up for LTFU patients = 12 months after last clinic visit before LTFU[b]				
2014	4,150	42	1.01 [0.75-1.37]	p=0.0029
2015	4,476	32	0.72 [0.51-1.01]	
2016	4,709	32	0.68 [0.48-0.96]	
2017	4,640	21	0.45 [0.30-0.69]	
2014-2017	17,976	127	0.71 [0.59-0.84]	
Sensitivity analysis 4: end of follow-up for lost to follow-up patients = date of last clinic visit before LTFU[b]				
2014	3,930	41	1.04 [0.77-1.42]	p=0.0026
2015	4,270	31	0.73 [0.51-1.03]	
2016	4,491	32	0.71 [0.50-1.01]	
2017	4,607	21	0.46 [0.30-0.70]	
2014-2017	17,298	125	0.72 [0.61-0.86]	
Sensitivity analysis 5: end of follow-up for HCV negative patients with continued follow-up in the cohort = date of last clinic visit[c]				
2014	4,024	41	1.02 [0.75-1.38]	p=0.0391
2015	4,309	31	0.72 [0.51-1.02]	
2016	4,463	32	0.72 [0.51-1.01]	
2017	3,591	21	0.59 [0.38-0.90]	
2014-2017	16,387	125	0.76 [0.64-0.91]	
Sensitivity analysis 6: end of follow-up for HCV negative patients with continued follow-up in the cohort = date of last negative HCV test[c]				
2014	3,865	41	1.06 [0.78-1.44]	p=0.8770
2015	3,772	31	0.82 [0.58-1.17]	
2016	3,350	32	0.96 [0.68-1.35]	
2017	1,861	21	1.13 [0.74-1.73]	
2014-2017	12,848	125	0.97 [0.82-1.16]	

Notes: In main analysis, [a]date of infection = midpoint between the last negative and the first positive HCV test when the interval was < 6 months, or the date of first positive HCV positive test minus 3 months when > 6 months; [b]end of follow-up for LTFU patients = 6 months after last clinic visit before LTFU; [c]end of follow-up for HCV negative patients with continued follow-up in the cohort = December 31st 2017. CI=Confidence Interval, py= Person-years, LTFU=Lost to follow-up.

Clinical Epidemiology Group of the ANRS CO4-FHDH

- **Scientific committee**: S Abgrall, L Bernard, E Billaud, F Boué, L Boyer, A Cabié, F Caby, A Canestri, D Costagliola, L Cotte, P De Truchis, X Duval, C Duvivier, P Enel, H Fischer, J Gasnault, C Gaud, S Grabar, C Katlama, MA Khuong, O Launay, L Marchand, M Mary-Krause, S Matheron, G Melica-Grégoire, H Melliez, JL Meynard, M Nacher, J Pavie, L Piroth, I Poizot-Martin, C Pradier, J Reynes, E Rouveix, A Simon, L Slama, P Tattevin, H Tissot-Dupont.
- ***COREVIH coordinating center***: **French Ministry of Health** (T Kurth, A Esclade), **Technical Hospitalization Information Agency, ATIH** (A Banaei).
- ***Statistical analysis center***: **UMRS 1136 INSERM et UPMC** (D Costagliola, *Principal investigator*, S Abgrall, S Grabar, M Choufany, M Guiguet, P Lakrout, E Lanoy, S Leclercq, L Lièvre, M Mary-Krause, H Selinger-Leneman, E Marshall, V Potard).
- **COREVIH:**

Paris area:

Corevih Ile de France Centre (**Paris-GH Pitié-Salpétrière** : E Caumes, C Katlama, O Benveniste, M A Valantin, R Tubiana, R Palich, A Simon, R Agher, C Blanc, L Lenclume, Y Dudoit, N Qatib, M Pauchard, D Beniken; **Paris-Hôpital Saint-Antoine** : K Lacombe, L Fonquernie, N Valin, M Brucker, M Sebire-Le Cam, J L Lagneau, J Lamarque, C Tran ; **Paris-Hôpital Tenon** : G Pialoux, R Calin, J Chas, M Hamidi, A Adda-Lievin, P Thibaut, M Mebarki).

Corevih Ile de France Est (**Hôpital Saint Louis** : J M Molina, C Lascoux, C Pintado, N de Castro, B Denis, W Rozenbaum, D Ponscarme, J Zeggagh, F ClaveL, A Aslan, M Lafaurie, J Gras, V Manda, G Liegeon, M Tateo, D Feyeux, G Hamet, C Colladant ; **Hôpital Lariboisière** : P Sellier, A Rami, M Diemer, L Azemar, M Parrinello, G Castor-Alexandre ; **CH d'Arpajon** : S Lamy ;**GHEF de Coulommiers** : B Devaux , S Tassi ; **GHEF de Marne la Vallée** : M Machado, P Simon, E Froguel, A Barrelet , O Senard ; **CH de Fontainebleau** D Cote, C Routier, H Roukas ; **CH de Melun** S Diamantis, N Vignier **; GHNE de Longjumeau** : O Banini ; **CHSF de Corbeil Essonnes** : A Chabrol, A Henn, T Gabas, O Son, N Aghe ; **CH de Bligny** : C Dupont, P Chardon ; **Hôpital Avicenne-Bobigny** : O Bouchaud, A Benmammar, L Traore ; **CHI Le Raincy-Montfermeil** : F Bidegain, L Mansouri ; **CHI André Grégoire-Montreuil** : M H Andre, G Fremont ; **Hôpital Jean Verdier** : J Bottero, C Palacios, F Mfutila, A Benmammar).

Corevih Ile de France Nord **(Hôpital Bichat-Claude Bernard** : Y Yazdanpanah, R Landmann C Mackoumbou-Nkoula, M Chansombat , C Godard, Z Julia, F Louni, A Ndiaye, L Loste, S Le Gac ; **CH Delafontaine-Saint-Denis** : M A Khuong-Josses, M Poupard, N Sayre, B Frison, C Charpentier, S Cossec ; **CH René DUBOS-Pontoise** : S Harent, L Blum, S Stegmann, G Grain ; **Hôpital Beaujon-Clichy** : B Fantin, A Villemant, A Lefort, V Zarrouk, V De Lastours, E Canoui, A Galy, V Honsel, A Becharef ; **CH de Gonesse** : D Seret Begue, D Troisvallets, A Becharef).

Corevih Ile de France Ouest (**CH de Mantes-la-Jolie** : F Granier, J J Laurichesse, B Montoya; **IHFB de Levallois –Perret** : D Giely, G Force; **CHI de Poissy** : C Veyssier-Belot, H Masson, Y Welker, C Leclerc ; **CH de Versailles** : A Greder-Belan, C Godin-Collet, D Bornarel ; **CHI de Meulan-les-Mureaux** : B Coudert, C Kakon, T Akpan, M Marcou ;**Hôpital Louis-Mourier** : H Duboc, E Mortier, A F Zeng; **Hôpital Ambroise Paré** : T Hanslik, J E Kahn, E Rouveix Nordon, S Greffe, S Trad, L Coutte, M, de Laroche, A Bauvois, A Freire Maresca, S Chenakeb ; **Hôpital Raymond-Poincaré** : C

Perronne, P de Truchis, H Lepinay ; **Hôpital Max Fourestier** : L El Mansouf, V Daneluzzi, V Manceron, E Mortier, G Bouteria ; **Hôpital Foch** : F Ackermann, E Fourn, C Majerholc, A Fadli ; **CH d'Argenteuil** : P Genet, F Caby, Bwifaq, J Gerbe).

Corevih Ile de France Sud (**Hôpital Cochin** :O Launay ; **Hôpital Européen Georges Pompidou** : L Weiss, J Pavie, D Batisse, M Karmochkine , M Meghadecha, ML Lucas, M Ptak, C Jung ; **Hôpital Hôtel Dieu** : J P Viard, D Salmon, L Slama, V A Letembet-Ippet, R Usubillaga, M Kalambay, R Ben Rayana, A Cros, M J Dulucuq, V Le-Baut, M P Pietri ; **Hôpital Necker-Enfants malades et Centre Médical de l'Institut Pasteur** : C Aguilar, F Alby-Laurent, K Amazzough, G Benabdelmoumen, P Bossi, G Cessot, C Charlier, P H Consigny, C Duvivier, K Jidar, E Lafont, F Lanternier, M Lecuit, E Pichard, J Leporrier, O Lortholary, C Louisin, J Lourenco, P Parize, B Pilmis, C Rouzaud, F Touam ; **Institut Mutualiste Montsouris** : M Gayraud, L Bodard ; **Hôpital Antoine-Béclère** : S Abgrall, I Kansau, V Chambrin, S Poirier ; **CHU Kremlin-Bicêtre** : C Goujard, S Jaureguiberry, Vittecoq A Castro Gordon, A Cheret, Y Quertainmont, E Teicher, O Derradji, M Bary, C Couzigou, J Y Liotier, M Merad, P Benoist, M Mole, F Churaqui, A S Keita ; **Hôpital Henri Mondor** : J D Lelievre, Y Levy, S Gallien, G Melica, W Vindrios, J L Lopez-Zaragoza, C Chesnel, R Ben Rayana ; **CHI DE Créteil** : I Delacroix, V Garrait, B Elharrar, E Krastinova, L Richier ; **CHI de Villeneuve-St-Georges** : O Patey S Dellion).

Outside Paris area :

Corevih de l'Arc Alpin (**CHU Grenoble Alpes** : P Leclercq, P Pavese, S Aggoun, S Dufresnes; **CH Alpes Leman** : D Leduc, S Megessier ; **CH d'Annecy Genevois** : V Vitrat, E Piet, A Valran; **CH de Chambery** : E Forestier, O Rogeaux, A Curiallet ; **CH de Thonon les Bains** : F Bissuel ; **Hôpital de Sallanches** : F Lange ;**CH Bourgoin Jallieu** : M Fabre; **CH Vienne** : A Bouaziz, L Adelaide ; **CH de Voiron :** C Henry-Ostian, M Guillaume).

Corevih Auvergne-Loire (**CHU de Clermont –Ferrand**: H Laurichesse, C Jacomet, E Goncalves ; **CHU de St Etienne** : P Berthelot, A Fresard, V Ronat ; **CHG Henri Mondor** : S Trouiller, I Masse-Chabredier, C Theis ; **CH Emile Roux** : O de Tauriac ; **CH de Roanne** : L Moiron, C Blanc ; **CH de Montluçon** : O Antoniotti, E Nehme ; **CH de Moulins-Yzeure** : A D Anthony Moumouni, Y Rousseau ; **CH Jacques Lacarin** : A M Milesi-Lecat, F Gourdon).

Corevih Bourgogne Franche-Comté (**CHU de Dijon** : L Piroth, P Chavanet, M Duong, M Buisson, A Waldner-Combernoux, S Mahy, A Salmon-Rousseau, M Blot, C Esteve, C François-Xavier, C Charles, S Gohier ; **Hôpital Pierre Bérégovoy-Nevers** : H Djerad, S Goteni; **CH de Sens :** A Creuwels ; **CHRU de Besançon** : C Chiroue, F Aubin C Drobacheff Thiébaut, A Foltzer, O Babre, E Chevalier ; **CH de Chalon-sur-Saône** : B Martha ; **CH de Mâcon** : J P Kristermann ; **Hôpital Nord Franche-Comté-Belfort** : V Gendrin, G Loriol **GH de la Haute-Saône-Vesoul** : C Merle**).**

Corevih Bretagne (**CHRU de Rennes** : P Tattevin, C Arvieux, M Revest, F Souala, S Patrat-Delon, M Baldeyrou, J M Chapplain, F Bénézit, M Dupont, A Maillard, F Lemaître, E Polard, J Vivent, J P Sinteff, T Jovelin, C Morlat, M Poisson, J P Sinteff ; **CH de Saint-Malo** : E Goarant, M Dupont ; **CH de Saint-Brieuc** : M Valence, R Buzele; **CHIC de Quimper** : P Perfezou, P Hutin, J P Talarmin, L Khatchatourian, J C Duthe ; **CH des Pays de Morlaix** : A Lamour, J B Euzen ; **CHRU La Cavale Blanche-Brest** : L de Saint-Martin, V Bellein, J C Duthé, S Briand-Feve ; **CHBA de Vannes** : Y

Poinsignon, G Corvaisier, M Gousseff, M Abdel Fattah, V Mouton Rioux, K Touboulic ; **CHBS de Lorient** : P Moreau, M Niault-Dollon, M F le Coz, O Luycx, A Lorléac'h, N Rouzic : **CH du Centre Bretagne** : J M Le Roux)

Corevih Centre-Val de Loire (**CHRU Tours** : L Bernard, O Bourgault, I Arnault, V Latkowski ; **CH Bourges**: A Maakaroun, Y Guimard, M Tissinie ; **CH Vierzon**: J Agbodjan ; **CH Chartres**: O Raffy, I Darasteanu, G Thomas, C Boulard ; **CH Dreux** : V Papillon, O Brasse, C Poirier; **CH de Châteauroux**: C Allais ; **CH de Blois et de Vendôme**: A Chekroun, V Cueille-Descarpentries; **CHR d'Orléans** : T Prazuck, L Hocqueloux, De Dieuleveult)

Corevih Grand-Est (**CHU de Strasbourg** : D Rey, M Partisani, V Martin, P Fischer ; **CHR Mulhouse Sud Alsace** : G Beck-Wirth, M Benomar ; **Hôpitaux civils-Colmar** : M Martinot, M Mohseni Zadeh, S Gravier, T Bonijoly, A Pachart ; **CHRU de Nancy** : B Hoen, M Delestan, M P Bouillon ; **CHI Emile Durkheim-Epinal** : M H Schuhamcher, I Beguinot ; **Polyclinique Saint-André-Courlancy** : C Strady ; **Hôpital Robert Debré – CHRU de Reims** : F Bani-Sadr, I Kmiec ; **CHR Metz-Thionville** : C Robert, P Muller, Z Cavelli, R Sibiescu, J Pouaha, M A Bucher ; **CH de Troyes**: P Chauveau-Jouve, S Pavel, L Fevre)

Corevih Hauts-de-France (**CH Tourcoing** : E Senneville, O Robineau, M Digumber, P Cornavin, M Lombard , N Decaux ; **CH de Compiègne** : A L Lecapitaine, M Tonnelier, A Bouras ; **CH de Creil :** N Landgraf, F Cordier ; **CH de Beauvais** : K Ghomari, R Barruet ; **CH de Lens** : H Bazus, E Aissi, A Legrain N Van Grunderbeeck, M Karas ; **CH de Boulogne-sur-Mer** : P Bataille ; **CHU d'Amiens** : J L Schmit, J P Lanoix, N Mathon, M Lombard ; **CH de Soissons** : A Hachemi ; **CH de Laon** : K Ainine ; **CH de Saint-Quentin** : Y Douadi)

Corevih Lyon-Vallée du Rhône (**Hospices civils de Lyon** : C Chidiac, M Plantier, N Danelon, S Degroodt, N Benmakhlouf , F Moncorge ; **Hôpital d'Instruction des armées Desgenettes -Lyon**: L Karkowski ; **CH de Bourg-en-Bresse** : N Canu, D Bouhour, P Granier, C Decouchou, E Racamier ; **Hôpital Nord-Ouest-Villefranche-sur-Saône** : G David, R Bricca ; **CVMV-Villeurbanne** : B Issartel ; **CH d'Ardèche Méridionale-Aubenas** : M A Arthus ; **CH d'Ardèche-Nord-Annonay** : J M Marc, E Legrand ; **Virages Santé-Lyon** : C Coudeyras, J M Livrozet ; **CH de Valence**: H Champagne, A Dureault, L Letranchant, C Reynaud, J Saison)

Corevih Normandie (**CHU de Caen :** P Goubin, S Dargere, R Verdon ; **CH de Saint Lô** : J Fouchard, C Briere, A Nicolle ; **CHI Elbeuf-Louviers-Val de Reuil** : L Chauffrey, D Theron ; **Hôpital Charles-Nicolle-Rouen** : F Caron, M Etienne, D Theron, S Plumecocq, G Unal ; **CH de Dieppe** : S Robaday Voisin ; **Hôpital Jacques Monod-Le Havre** : N Elforzi, A Vandendriessche, C Guillois)

Corevih Nouvelle-Aquitaine (**CH d'Angoulème** : A Riché, S Males, M Grosset, E C Ngo Bell, G Arnou ; **CH de Chatellerault** : G Romero De Avila, A Elsendoorn ; **CH de Jonzac-Saintonge-Saintes- Saint-Jean d'Angely** : T Pasdeloup ; **CH de Niort** : S Sunder, A Dos Santos, V Goudet, K Schepers, P Gougeon ; **CH de Royan** : P Mottaz; **CHI du Pays de Cognac** : S Hebert ; **CHU de Poitiers** : F Cazenave-Roblot, G Le Moal ; **CHU Dupuytren – Limoges** : J F Faucher, C Genet, J Pascual ; **G H de La Rochelle** : F Roblot, G Le Moal, G Beraud, M Catroux, D Plainchamp; **CH de Brive** : B Abraham)

Corevih Occitanie (**CH d'Albi** : J M Merault, M Chauveau, M A Laffont **; CH d'Alès** : D Bastide, T Fraisse, A Soualah **; CH de Bézier** : E Oziol, M L Casanova **; CH de Bigorre – Tarbes** : C Dingremont, Y Leveneur, A Soto, B Marchou **; CH de Cahors** : S Sire, V Remy **; CH Jacques Puel – Rodez** : F Abdo, M Loriette, S Ray, B Albinet, H Frahier, B Guerin **; CH St Clair – Sète** : **; CH St-Jean – Perpignan** : H Aumaitre, M Saada, F Roustant, L Pinheiro **; GHU Caremeau – Nîmes** : A Sotto, I Rouanet, R Doncesco **; Hôpital Gui de Chauliac – Montpellier** : J Reynes, A Makinson, F Sebastiani, C Fernandez, C Tramoni, C Blot **; Hôpital J. Ducuing – Toulouse** : F Gaches, D Garipuy, M Michaud, A Bicart-See, E Bonnet, F Trognko **; Hôpital Purpan - CHU de Toulouse :** P Delobel, P Lansalot-Matras, M Marcel, C Rastoll, S Lagarrigue, A Frelat)

Corevih PACA Est (**CH d'Antibes** : A Lanteri, E Denis, S Tempesta , A Vallejo ; **CH de Nice** : P Pugliese, V Mondain, E Cua, N Oran, I Touitou ; **CH de Cannes** : M Vassalo, N Oran, E Escach Wessels ; **CH Général de Grasse Clavary** : P Y Dides ; **CHI de Fréjus** : P Del Giudice, T Hubiche, V Courbon ; **CH de Draguignan** : A Boumallassa, A Mothes, C Girod-Jouffroy, S Gobin).

Corevih PACA Ouest-Corse (**Marseille-Hôpital de la Conception**: P Enel, R Sambuc, MS Antolini-Bouvenot, P Druart, L Meddeb, I Ravaux, A Menard, C Tomei, C Dhiver, H Tissot-Dupont; **Marseille-Hôpital Nord**: J Moreau, S Mokhtari, MJ Soavi, V Thomas; **Marseille-Hôpital Sainte-Marguerite**: I Poizot-Martin, S Bregigeon, O Faucher, V Obry-Roguet, AS Ritleng, N Petit; **Marseille-Centre pénitentiaire des Baumettes**: C Bartoli, JM Ruiz, D Blanc; **CH d'Aix-En-Provence**: T Allegre, M Sordage, JM Riou, C Faudon; CH d'Avignon: B Slama, H Zerazhi, O Boulat, S Chebrek, M Beyrne; **CH de Digne Les Bains**: P Granet Brunello; **CH de Gap**: L Pellissier, D Bonnabel; **CH de Martigues**: R Cohen Valensi, B Mouchet, G Mboungou; **CHI de Toulon**: A Lafeuillade, E Hope-Rapp, G Hittinger, G Philip, V Lambry).

Corevih Pays de la Loire (**CHU de Nantes** : F Raffi, C Allavena, E Billaud, D Boutoille, C Deschanvres, B Gaborit, P Le Turnier, V Reliquet, S Sécher, M Cavellec, E Paredes, A Soria ; **CH de Saint-Nazaire** : S Hily, C Michau, N Pouhaut ; **CHU d'Angers** : P Abgueguen, V Dubée, V Delbos, V Rabier, Y M Vandamme, H Cormier, D Sanderink, R Mahieu, M de La Chapelle, P Fialaire, S Rehaiem ; **CH de Cholet** : S Monnereau, J P Breux, L Seguin ; **CH de Laval** : R Vatan, F Dangeul-Potier ; **CH du Mans** : H Hitoto, S Blanchi, N Crochette, J B Laine, L Perez, F Dangeul ; **CH de La Roche-sur-Yon** : P Perre, S Leautez, D Merrien, T Guimard, O Bollengier Stragier, M Morrier, D Boucher, P Point, J L Esnault, H Durand, A Pegeot, L Laine, T Jovelin, H Pelerin).

Overseas:

Corevih Guadeloupe (**CHU de Pointe-à-Pitre** : P M Roger, I Fabre, I Lamaury, B Tressieres, E Duvallon, K Samar ; **CH de Basse-Terre** : N Baillet, F Boulard, M C Receveur, C Desfontaines ; **CH Saint-Martin**: : S Paucod, C Clavel, C Daniel, V Walter),

Corevih Guyane (**CHG de Cayenne**: M Nacher, S Soyon, K Verin, L Saint-Louis, K Bienvenu, B Guarmit, A Tricoche).

Corevih Martinique (**CHU de Fort-de-France**: A Cabie, S Abel, L Cuzin, S Pierre-François, K Guitteaud, J M Turmel, K Rome, D de Majoubert, J Bavay).

Corevih Océan Indien (**St Denis-CHU Félix Guyon** : M P Moiton, C Ricaud, B Kuli, C Gaud, M Lagrange-Xelot, S Hoang, G Wartel, R Rodet, C Sautron, I Rigollier ; **St Pierre-GH Sud Réunion** : P Poubeau, R Manaquin Y Koumar, C Duronea; **CH de Mayotte :** S Permal, M Jean).

A Dureault, L Letranchant, C Reynaud, J Saison
